# Feasibility and sustainability of dietary surveillance, Bosnia and Herzegovina

**DOI:** 10.2471/BLT.18.227108

**Published:** 2019-03-25

**Authors:** Selma Gicevic, Emir Kremic, Teresa T Fung, Bernard Rosner, Edin Sabanovic, Walter C Willett

**Affiliations:** a Department of Nutrition, Harvard T.H. Chan School of Public Health, 665 Huntington Ave, Boston, Massachusetts, MA 02115, United States of America (USA).; b Institute for Statistics of the Federation of Bosnia and Herzegovina, Sarajevo, Bosnia and Herzegovina.; c Department of Nutrition, Simmons University, Boston, USA.; d Department of Medicine, Brigham and Women's Hospital, Boston, USA.; e Agency for Statistics of Bosnia and Herzegovina, Sarajevo, Bosnia and Herzegovina.

## Abstract

National dietary surveillance systems are necessary for monitoring people’s intake of foods and nutrients associated with health and disease, and for implementing national and global dietary goals. However, these systems do not exist in many low- and middle-income countries. The development of a model of dietary surveillance for Bosnia and Herzegovina, described here, provides insights into the feasibility and sustainability of dietary surveillance systems in resource-constrained settings and illustrates the challenges involved. In 2016, a year-long dietary survey was initiated in collaboration with the country’s Institute for Statistics using a subsample of households that participated in the 2015 national Household Budget Survey. Interviewers collected lifestyle, anthropometric and health data and participants answered two 24-hour dietary recall questionnaires. The survey included a representative sample of 853 participants and was performed efficiently by a small team of highly motivated, well-trained staff. Conducting a high-quality dietary survey was found to be feasible despite constrained resources. In addition, the ability to link dietary intake and regular household survey data provided an effective way of associating dietary variables with socioeconomic determinants of health. This dietary survey, the first conducted by an official institution in Bosnia and Herzegovina, represents an important starting point for building a sustainable nutritional surveillance system for the country. The cost–effective, low-burden approach to dietary surveillance described here could be applied in other low- and middle-income countries, many of which already carry out regular economic surveys.

## Résumé

Les systèmes nationaux de surveillance alimentaire sont nécessaires pour contrôler la consommation par la population des aliments et nutriments liés à la santé et aux maladies, mais aussi pour réaliser les objectifs nationaux et internationaux relatifs à l'alimentation. Or, il n'existe aucun système de la sorte dans nombre de pays à revenu faible et intermédiaire. La conception d'un modèle de surveillance alimentaire pour la Bosnie-Herzégovine que nous décrivons ici permet de mieux comprendre la faisabilité et la durabilité des systèmes de surveillance alimentaire dans les régions aux ressources limitées et illustre les défis à relever. En 2016, une enquête sur l'alimentation a été menée pendant une année, en collaboration avec l'institut national de la statistique de Bosnie-Herzégovine, auprès d'un sous-échantillon de ménages qui avaient pris part à l'enquête nationale de 2015 sur le budget des ménages. Les enquêteurs ont collecté des informations sur le mode de vie, des données anthropométriques et des données de santé, et les participants ont répondu à deux questionnaires fondés sur le rappel de leur consommation au cours des dernières 24 heures. Cette enquête a inclus un échantillon représentatif de 853 participants et a été efficacement menée par une petite équipe de personnes extrêmement motivées et bien formées. La réalisation d'une enquête de grande qualité sur l'alimentation s'est avérée faisable, malgré des ressources limitées. Par ailleurs, la possibilité de relier les données concernant les consommations alimentaires et les données des enquêtes régulièrement menéessur les ménages a fourni un moyen efficace d'associer les variables relatives à l'alimentation avec les déterminants socioéconomiques de la santé. Cette enquête sur l'alimentation (la toute première menée par un institut officiel en Bosnie-Herzégovine) constitue un bon point de départ pour l'élaboration d'un système de surveillance nutritionnelle durable pour le pays. L'approche de la surveillance alimentaire décrite ici, qui s'est avérée rentable et relativement peu pesante, pourrait être appliquée dans d'autres pays à revenu faible et intermédiaire, dont la plupart réalisent déjà régulièrement des enquêtes économiques.

## Resumen

Los sistemas nacionales de vigilancia de la alimentación son necesarios para controlar la ingesta de alimentos y nutrientes asociados con la salud y las enfermedades, así como para alcanzar los objetivos alimentarios nacionales y mundiales. Sin embargo, estos sistemas no existen en muchos países de ingresos bajos y medios. La elaboración de un modelo de vigilancia alimentaria para Bosnia y Herzegovina, que se describe aquí, permite comprender mejor la viabilidad y la sostenibilidad de los sistemas de vigilancia alimentaria en entornos con recursos limitados e ilustra los problemas que se plantean. En 2016, se inició una encuesta alimentaria de un año de duración en colaboración con el Instituto de Estadística del país a partir de una submuestra de hogares que participaron en la Encuesta nacional de presupuestos familiares de 2015. Los entrevistadores recopilaron datos de estilo de vida, antropométricos y de salud y los participantes respondieron a dos cuestionarios de recordatorio alimentario de 24 horas. La encuesta incluyó una muestra representativa de 853 participantes y fue realizada eficazmente por un pequeño equipo de personal altamente motivado y bien capacitado. La realización de una encuesta alimentaria de alta calidad resultó factible a pesar de la escasez de recursos. Además, la capacidad de vincular la ingesta alimentaria con los datos de las encuestas periódicas en los hogares era una manera eficaz de asociar las variables alimentarias con los factores socioeconómicos determinantes de la salud. Esta encuesta sobre la alimentación, la primera realizada por una institución oficial de Bosnia y Herzegovina, representa un punto de partida importante para la creación de un sistema sostenible de vigilancia alimentaria para el país. El enfoque rentable y de baja carga de la vigilancia alimentaria que aquí se describe podría aplicarse en otros países de ingresos bajos y medios, muchos de los cuales ya llevan a cabo encuestas económicas periódicas.

## ملخص

تعد أنظمة الرقابة الغذائية الوطنية ضرورية لرصد استهلاك الأشخاص للأطعمة والمواد الغذائية المرتبطة بالصحة والمرض، ولتنفيذ الأهداف الغذائية الوطنية والعالمية. ومع ذلك، إلا أن هذه النظم لا تتواجد في العديد من البلدان منخفضة الدخل والبلدان متوسطة الدخل. إن تطوير نموذج للرقابة الغذائية في البوسنة والهرسك، الذي نتناوله بالشرح هنا، يقدم نظرة ثاقبة على مدى جدوى واستدامة نظم الرقابة الغذائية في ظل أوضاع محدودة الموارد، ويوضح التحديات التي تنطوي عليها. في عام 2016، تم إطلاق مسح غذائي لمدة عام بالتعاون مع معهد الإحصاءات بالدولة، باستخدام عينة فرعية من الأسر التي شاركت في مسح ميزانية الأسرة لعام 2015. قام المشرفون على المقابلات الشخصية بتجميع بيانات حول نمط الحياة والبيانات الأنثروبومترية والصحية، وأجاب المشاركون على استبيانين للتذكير الغذائي على مدار 24 ساعة. وشمل المسح عينة تمثيلية من 853 مشاركًا وتم تنفيذه بفعالية بواسطة فريق صغير من الموظفين المتحمسين للغاية والحاصلين على تدريب جيد. تبين أن إجراء مسح غذائي عالي الجودة أمر ممكن رغم الموارد المحدودة. بالإضافة إلى ذلك، فإن القدرة على الربط بين الاستهلاك الغذائي وبيانات المسح الأسري المعتاد، قدمت وسيلة فعالة لربط المتغيرات الغذائية مع المحددات الاجتماعية والاقتصادية للصحة. هذا المسح الغذائي هو الأول من نوعه الذي تقوم به مؤسسة رسمية في البوسنة والهرسك، وهو يمثل نقطة انطلاق مهمة لبناء نظام رقابة غذائية مستدامة للبلد. إن أسلوب الرقابة الغذائية الذي يتميز بالفعالية من حيث التكلفة والعبء المنخفض، والذي نقوم بوصفه هنا، يمكن تطبيقه في البلدان الأخرى ذات الدخل المنخفض، والدخل المتوسط والكثير منها بالفعل يقوم بإجراء مسوحات اقتصادية معتادة.

## 摘要

国家膳食监测系统对于监测人们摄入的食物和营养物质与健康和疾病状况之间的关联，以及实施全国性和全球性膳食目标是十分必要的。然而，许多低收入和中等收入国家并没有此类系统。本文描述了波斯尼亚和黑塞哥维那膳食监测系统的发展，提供了膳食监测系统在资源有限的环境下可行性和可持续性的见解，并就所涉及的挑战予以说明。2016 年，与该国统计研究所合作开展了一项为期一年的膳食调查，并使用了参与 2015 年全国家庭预算调查的家庭子样本。采访者收集了生活方式、人体测量和健康数据，同时参与者回答了两份 24 小时膳食回顾调查问卷。本调查纳入了 853 名参与者的代表性样本，并由一小组积极性高、训练有素的员工团队高效完成。尽管资源有限，但进行高质量的膳食调查仍切实可行。此外，能够将膳食摄入量与定期的家庭调查数据相联系为将膳食变量与健康的社会经济决定因素相联系提供了一种有效方法。此项膳食调查——首次由波斯尼亚和黑塞哥维那的官方机构进行——是该国建立可持续营养监测系统的重要基础。本文所述的低成本、低负担的膳食监测方法可应用于其他低收入和中等收入国家，其中许多国家已开展定期经济调查。

## Резюме

Национальные системы надзора за качеством питания необходимы для мониторинга употребления людьми пищевых продуктов и питательных веществ в связи с проблемами со здоровьем или заболеваниями, а также для реализации национальных и глобальных целей в области питания. Однако во многих странах с низким и средним уровнем доходов таких систем не существует. Разработка модели системы надзора за качеством питания в Боснии и Герцеговине, описанная в данном документе, позволяет достоверно представить практическую осуществимость и перспективность систем надзора за качеством питания в условиях ограниченных ресурсов и наглядно демонстрирует связанные с ними проблемы. В 2016 году был проведен годичный опрос для определения характера питания населения. Опрос проводился в сотрудничестве с национальным Институтом статистики на подвыборке семей, участвовавших в национальном опросе по семейному бюджету в 2015 году. Лица, проводившие опрос, собирали данные об образе жизни, антропометрические показатели и данные о здоровье, а участники опроса должны были по памяти дать ответы на две анкеты о своем питании за последние 24 часа. Опросом была охвачена репрезентативная выборка из 853 участников. Опрос проводился небольшой группой заинтересованных и квалифицированных сотрудников. Проведение качественного опроса для определения характера питания населения оказалось возможным, несмотря на ограниченность ресурсов. Кроме того, возможность связать данные рациона питания с данными стандартного опроса о состоянии семьи позволила установить связь между переменными, связанными с качеством питания, и социально-экономическими детерминантами здоровья. Данный опрос для определения характера питания (первое мероприятие подобного рода, проведенное государственным учреждением в Боснии и Герцеговине) является важной отправной точкой для создания надежной системы надзора за качеством питания в стране. Экономичный и сопряженный с небольшими нагрузками подход к надзору за качеством питания, описанный в данном документе, может применяться и в других странах с низким и средним уровнем доходов, многие из которых уже проводят регулярные экономические обследования.

## Introduction

Diet and its associated health conditions are the most important modifiable risk factors for morbidity and mortality globally. The influence of diet on health is greater than that of tobacco, alcohol and drug use, and unsafe sex combined.^1^^,^^2^ Moreover, there is evidence that even small dietary modifications can improve nutrition and health.^3^^–^^6^ The availability of accurate, individual data on a population’s diet is critical for developing dietary policies and programmes and for monitoring their implementation, both at a national (e.g. adherence to dietary guidelines) and a global level; for example, to achieve targets such as the sustainable development goal 2 to “end hunger and all forms of malnutrition.”^7^ Nevertheless, recent reviews of dietary surveillance worldwide reveal that data are scarce in many parts of Africa, Asia and eastern and south-eastern Europe.^8^^–^^10^

The dietary surveillance systems developed in high-income countries, such as the National Health and Nutrition Examination Survey (NHANES) in the United States of America and the United Kingdom’s National Diet and Nutrition Survey are expensive, complex and burdensome.^9^^,^^11^^–^^13^ Typically they require a large sample, a team of experts and substantial investment in equipment and software. However, many countries with resource constraints are left without these crucial decision-making aids for promoting population health. Without population data on food and nutrient intake, policy-makers risk making the wrong decisions or may ignore the problem of inadequate food systems and diet altogether. The United Nation’s Secretary-General’s Independent Expert Advisory Group on a Data Revolution for Sustainable Development has called for urgent action to enhance national capacity for efficient data collection to bridge the gap between developed and developing countries.^14^ Although the so-called data revolution taking place globally as a result of rapid technological development provides both a challenge and an opportunity, some countries have been left out due to a lack of resources, knowledge, capacity or opportunity.^14^ In 2017, the United Nation’s World Data Forum highlighted the need to modernize national statistics systems and to expand them to include data domains currently beyond the scope of official statistics.^15^ In particular, the accurate analysis of diet in disadvantaged population groups (e.g. low-income groups, single-parent families and minorities) depends on linking dietary surveillance systems to existing demographic and socioeconomic data.

Seeking new ways to obtain good-quality dietary data in countries with financial and workforce constraints is, therefore, an important goal for researchers. Here, we describe our experience with developing dietary surveillance for Bosnia and Herzegovina and consider the challenges associated with establishing a feasible and sustainable dietary surveillance system in resource-constrained settings.

## Developing the system

Bosnia and Herzegovina is a middle-income country in south-east Europe that has been struggling with an inadequate dietary surveillance system and constrained resources because its social and economic development has been hindered by complex inter-ethnic and political problems since the breakup of Yugoslavia in 1992.^16^^,^^17^ The most recent census in 2013 recorded a population slightly over 3.5 million.^18^ In 2012, one quarter of children and one half of adults were overweight or obese, half of all deaths were due to stroke or heart disease, type-2 diabetes was on the rise, and a quarter of women of reproductive age were at risk of iron-deficiency anaemia.^19^^,^^20^ However, data on diet were sparse. Apart from the food balance sheets produced by the United Nations’ Food and Agriculture Organization, the only sources of data on food and diet in Bosnia and Herzegovina were the 2015 Household Budget Survey, which included food expenditure data,^21^ and occasional population health surveys, which included a short-form, food-frequency questionnaire.^20^ Both these data sources were heavily dependent on donor funding and external expertise. We were unable to identify any food consumption surveys in the country that included data from individuals. In response, the Institute for Statistics of the Federation of Bosnia and Herzegovina and the Harvard T.H. Chan School of Public Health in the United States of America decided to conduct a pilot diet survey in the country between November 2016 and November 2017.

### Considerations

A new model of dietary surveillance is needed for countries with constrained resources: it should provide a quick and affordable assessment of diet without sacrificing data quality. Box 1 presents what such a model should consider at a minimum.

Box 1 **Considerations for a model of dietary surveillance for countries with constrained resources**

- Cover a representative sample of the population, ideally a nationally representative sample would provide results generalizable to the entire population.
- Collect data from individuals (in contrast to household spending surveys or food balance sheets) and link to data from existing surveys (e.g. economic surveys) so that diet can be analysed in terms of demographic and socioeconomic variables.
- Collect data continuously or, at least, periodically (e.g. four-yearly) to enable trend analysis.
- Use dietary assessment methods that are standardized and harmonized with those in other countries to permit cross-country comparisons.^13^
- Analyse both food and nutrient intake to identify nutrients that are consumed either inadequately or in excess;
- Focus on the “usual intake” of the population, not on intake on any one day.
- Provide data on the intake distribution (i.e. on the prevalence of inadequate and excessive intake) in addition to the mean intake (the primary goal).

Studies have identified several considerations that should be taken into account when setting up a national or global dietary and nutrition surveillance system: (i) demand for information; (ii) the institutional base for dietary surveys; (iii) resource needs (i.e. cost and staff time); (iv) personnel capacity; (v) technical capacity; (vi) the diet assessment method; (vii) a country-specific food composition database; and (viii) data user participation.^13^^,^^22^ Table 1 details how some of these considerations were tackled in establishing a dietary survey for Bosnia and Herzegovina and briefly describes future challenges.

**Table 1:** Considerations in the development of dietary surveillance, Bosnia and Herzegovina, 2016–2017

Component	Initial challenge	Strategy	Resources	Future challenges
Institutional base	No existing institutional base for survey data	(i) The Institute for Statistics,^a^ a government body independent of policy-makers,^b^ was selected as the institutional base; (ii) the government of Bosnia and Herzegovina approved the pilot survey as part of the Institute’s 2017 annual plan	(i) The Institute for Statistics provided access to detailed data in the master sampling frame used in previous surveys and to infrastructure and local connections (e.g. regional offices, telephones, printing and links to police, municipal authorities and local media); (ii) one local staff member provided by an academic partner trained to become a local contact for future dietary surveys; (iii) an academic partner provided financial resources to cover interviewer fees; (iv) clear protocols and operational procedures were developed for the survey	The government should include a dietary survey in routine data collection (e.g. four-yearly)
**Resource utilization**				
Sample	Large sample size needed for traditional dietary surveys	(i) Adopt a multiple-stage, cluster sampling approach; (ii) use the minimum sample size required for monitoring dietary intake in the population; (iii) use the minimum sample to achieve a precision of 5 to 10% for the sample means of each nutrient; (iv) employ existing local expertise, when possible	(i) Sampling frame for latest Household Budget Survey in 2015 used; (ii) sample design performed by the Household Budget Survey statistician	Strategies needed to reach young male participants
Staff	Large number of technical staff needed for traditional dietary surveys	Employ a small but efficient team, comprising: (i) a statistician; (ii) a supervisor; (iii) a nutritionist and dietitian; (iv) a communications officer; (v) an administrative and data clerk; and (vi) a technical support assistant	(i) Statistician^c^ (3 days; 0.02 FTE^d^) for sample design and calculating sampling weights; (ii) supervisor (0.28 FTE^d^) for survey design (20 days), team-leading (24 days), fieldwork oversight (12 days) and interviewer training (14 days); (iii) nutritionist and dietitian (0.36 FTE^d^) for assigning quantities and food codes in Diet Assess (15 minutes per 24-hour recall interview),^23^ dealing with missing and implausible values (5 days), SAS code (SAS Institute, Cary, USA) development (5 days), and survey development and report writing (10 days); (iv) communications officer^c^ (0.03 FTE^d^) for proof-reading and desktop publishing of the report (7 days) and coordinating data dissemination to data users and the general public; (v) administrative and data clerk^c^ (0.04 FTE^d^) for preparing and sending letters (5 days) and liaising with the public (4 days); and (vi) technical support assistant^c^ (0.09 FTE^d^) for developing and testing CAPI–CATI questionnaires (10 days) and technical troubleshooting (12 days); (vii) total staffing requirements were 0.66 FTE^d^ at the managerial level and 0.95 FTE^d^ for support staff (including interviewer time)	(i) The supervisor’s and nutritionist’s job profiles should be kept at the Institute for Statistics to achieve sustainability; (ii) alternatively, descriptive nutrient-based analyses of dietary data could be performed for the Institute for Statistics by the Diet Assess developers (as part of the service provided to all purchasers of Diet Assess software)
Interviewers	(i) Large number of interviewers needed; (ii) poor retention	(i) Focus on quality rather than quantity when selecting interviewers; (ii) interviewer profile: high level of motivation, computer literate, vehicle owner, resident in sample household cluster, assertive and determined personality, ability to understand the need for high data quality, and culturally sensitive and responsive to a participant’s adverse circumstances, while keeping a professional distance	(i) Four interviewers needed (230 participants each; 0.79 FTE^d^ in total); (ii) 2–3 reserve interviewers; (iii) individuals affiliated with the Institute for Statistics (either employees or members of an interviewer pool hired externally for field surveys) were preferred; (iv) our four interviewers were highly educated, computer literate, female and aged 30–40 years; (v) three male interviewers and one female opted out for different reasons (e.g. complex methodology, poor computer skills and a lack of assertiveness)	(i) Consider employing interviewers on a full-time basis in the Institute for Statistics and train them for multiple surveys; (ii) the interviewer’s gender may be important for some survey locations
Travel cost	High travel costs for fieldwork	(i) Select interviewers who reside within sample household clusters; (ii) ensure cluster is within a 90-minute drive for each interviewer; (iii) select one male and one female from each household whenever possible to minimize travel costs	None	It may be difficult to find interviewers with the desired characteristics for some clusters
**Data collection and analysis**				
Collection	Slow, paper-based data collection and compilation system	Use CAPI–CATI approach for data collection, with the option to collect data offline in areas without an internet connection	(i) CAPI–CATI software: our pilot study used Qualtrics software with an offline survey application for Android mobile phones;^24^ (ii) each interviewer was provided with a tablet computer; (iii) interviewers used a 3G wireless connection from their homes and regional offices	(i) Find a reliable offline survey application – data were occasionally lost during transfer with the application used in our pilot survey; (ii) find a simple way of automatically coding 24-hour recall questionnaire responses
Storage	Data stored in separate databases	(i) Integrate data from the 2015 Household Budget Survey with dietary survey data; (ii) use a subsample of respondent households from the Household Budget Survey for our dietary survey to enable data integration and analysis, such that diet can be linked to socioeconomic indicators and an individual’s dietary intake can be compared with household food consumption; (iii) this approach could reduce interviewer transport costs	None	(i) Update the sampling frame used for household budget surveys, which has become outdated due to political constraints; (ii) ensure a good response rate in urban areas; (iii) avoid overuse of the sampling frame, which may result in a high respondent burden and a high nonresponse rate; (iv) consider incentives for survey participation^e^
Analysis	(i) Complex nutrient-based analysis required; (ii) lack of a local food composition database	(i) Procure software for nutrition assessment linked to the local Balkan food composition database; (ii) train local staff to use the software	(i) Diet-assessment software and a food composition database – our dietary study used Diet Assess and the Balkan food composition database;^25^ (ii) one trained analyst to code food items, assign portion sizes and run basic descriptive statistics using SAS codes; (iii) food atlas for estimating portion sizes; (iv) SAS codes for analysis	None

CAPI: computer-assisted personal interviewing; CATI: computer-assisted telephone interviewing; FTE: full-time equivalent, SAS: statistical analysis system USA: United States of America.

^a^ The Institute for Statistics of the Federation of Bosnia and Herzegovina is one of three official statistics agencies in the country.

^b^ The Cape Town Global Action Plan for Sustainable Development Data, launched informally at the First United Nations World Data Forum in 2017, emphasized the modernization and strengthening of national statistical offices, including expanding their domains beyond data traditionally collected, so they could act as coordinators and strategic leaders for tracking progress on Sustainable Development Goals.^15^

^c^ Existing skilled personnel available at national statistics agencies.

^d^ The full-time equivalent (FTE) is the number of full-time equivalent jobs, which is derived from the total number of hours worked by the worker, divided by the average number of hours worked annually in a full-time job.^26^

^e^ Survey participants could be encouraged to participate in repeat surveys by receiving grocery vouchers, which could be provided free by a major supermarket chain as part of its social responsibility agenda.

### Institutional base

The lack of a natural institutional base, or home, for dietary surveys that is independent of policy-makers was identified as a key challenge.^13^^,^^22^ In Bosnia and Herzegovina, we asked the Institute for Statistics of the Federation of Bosnia and Herzegovina (a government body that is also an independent producer of official data), if it would take charge of implementing dietary surveys.^27^ Subsequently, the government approved the inclusion of a pilot diet survey in the Institute’s annual plan for 2017. The survey relied on the institute’s existing infrastructure, including staff, regional offices, telephones, printing facilities and links with municipal authorities, the police and local media. The institute’s involvement also provided access to the sampling frame used in previous surveys and enabled dietary data to be linked to other survey data at the individual level. One of the authors, who was a full-time nutrition scientist at the institute, underwent professional training at the Harvard T.H. Chan School of Public Health and, thereafter, developed the survey protocol, trained survey interviewers, supervised fieldwork, analysed data and prepared reports. The lead statistician at the Agency for Statistics of Bosnia and Herzegovina was responsible for the sample design. The study protocol was approved by the ethics committees of both the Bosnian Public Health Institute and the Harvard T.H. Chan School of Public Health’s Office of Human Research Administration. In addition, our approach was in accordance with the United Nations’ World Data Forum’s recommendations on modernizing national statistics systems and on expanding their domains beyond the data traditionally collected.^15^

### Resource utilization

The large sample size, the high cost of travel for interviewers and the large team of experts traditionally required to implement dietary surveys were additional challenges. To minimize sampling costs, we drew a cluster sample of households from the sampling frame used for the 2015 Household Budget Survey. The sample was nationally representative and comprised a two-stage, stratified random sample of households: (i) first, a sample of enumeration units (i.e. primary sampling units) was selected, with each unit having an equal probability of selection; and (ii) second, a sample of households (i.e. secondary sampling units) was drawn by applying Bethel’s optimal allocation algorithm using data for 24 variables from the previous household budget survey across strata.^28^ Restricting our selection of households to respondents in the 2015 Household Budget Survey enabled us to link dietary and socioeconomic data. We aimed for the minimum sample that was large enough to achieve a precision of 5 to 10% for the mean intake of each nutrient of interest. For the majority of nutrients, a sample of 100 to 250 individuals was sufficient, assuming a single day of data per person (vitamin A required a larger sample because of high within-person variability in intake).^29^ The European Food Safety Agency recommends including at least 130 individuals in each subgroup of interest (e.g. 130 males and 130 females in each age group).^30^ Consequently, a sample of around 1000 individuals was sufficient to provide high precision for the survey overall and reasonable precision for subgroups defined by age, gender, type of residence and other characteristics.

First, we selected primary sampling units that were within 90 minutes’ travel from the interviewer’s residence; then, we randomly selected households within these clusters. For methodological and financial reasons, we excluded children and adolescents, pregnant and breastfeeding women and elderly people with cognitive decline (each of these groups requires a different data collection protocol and a separate data analysis).^30^ Eligibility was determined using presurvey questions. Finally, we chose a sample size of 980 households on the assumptions that two people could be interviewed in around 50% of households and that the nonresponse rate would be around 40%.^31^

To minimize fieldwork costs, the first eligible male and female in each household were interviewed whenever possible. However, we reached the quota for female participants aged 60 years and older within 9 months, whereas young male participants were underrepresented. In the last 3 months of the survey (i.e. September to November 2017), we focused on young male participants to obtain sufficient numbers in each sex and age subgroup. We obtained written informed consent from each participant at recruitment. After three unsuccessful contact attempts, a household was regarded as unavailable. The refusal rate was the percentage of households that were successfully contacted but refused to participate. Subsequently, appropriate sampling weights were applied to adjust for design and participation effects.

### Personnel capacity

Local capacity was built among staff already employed by the Institute for Statistics. In addition to the nutritionist, a statistician and an information technology technician were available part time for sample design, calculating weightings, online questionnaire development and testing, and technical troubleshooting. The dietary and ancillary data collection protocol was developed by synthesizing the best practice of NHANES and the European Food Safety Agency.^11^^,^^30^ The protocol provided detailed instructions for interviewers on contacting participants, keeping records, conducting both dietary interviews (with particular attention to the multiple-pass method)^32^ and use of the food atlas.^33^ The protocol developed could be used in the next survey and passed on to any new members of staff. Interviewers were carefully selected from the Institute’s existing roster using predefined criteria and underwent training. The key requirements were: (i) a high level of motivation; (ii) computer literacy; (iii) ownership of a vehicle with the ability to travel within a 90-minute radius of their place of residence; (iv) an assertive personality; (v) an understanding of the importance of high data quality; (vi) cultural sensitivity; and (vii) professional demeanour. The funds required for implementing the dietary study were covered by the Institute for Statistics’ budget and there was no need to rely on donor funding (Box 2). Our approach was markedly different from previous practice in post-war Bosnia and Herzegovina, when donor-funded activities might have artificially inflated the cost of surveillance because costs were often determined by the amount of external funding, rather than by local needs and economic conditions.

Box 2 **Estimated costs, national dietary survey, Bosnia and Herzegovina, 2016–2017**

**Staff members:^a,b^**

Nutritionist (0.36 FTE): € 4320

Supervisor (0.28 FTE): € 2520

Statistician (0.02 FTE): € 240

Interviewers (0.79 FTE): € 7110

Support staff (0.16 FTE): € 1152

**Software: € 5000^c^**

**Travel costs for interviewers: € 3240^d^**

**Printing: € 300**

**Total: € 23 882**

FTE: full-time equivalent.^a^ The FTE is the number of full-time equivalent jobs, which is derived from the total number of hours worked by the worker, divided by the average number of hours worked annually in a full-time job.^26^^b^ We based staff members cost on net pro-rata annual pay.^c^ Includes software licence fee for government agencies and ongoing support from the licence provider.^d^ Assuming interviewers travel by car within a 90-minute radius of their place of residence, the total estimated distance travelled was 32 000 km with an estimated fuel consumption of 9 L/100km, giving a total fuel requirement of 2880 L.

### Diet assessment method

Selecting the most appropriate diet assessment method was another challenge because no single method is optimum for all purposes: for example, the best method for nutrition counselling may not be best for dietary surveillance or for evaluating the relationship between diet and disease. Inevitably, the choice of method involves a trade-off between statistical precision, the period covered, the level of detail and cost.^34^^,^^35^ For our study, interviewers used the five-step, multiple-pass method based on 24-hour dietary recall to obtain dietary data on two occasions:^32^ a household interview was followed by a phone call 4 to 30 days later. This approach is regarded as the preferred survey method for obtaining a good level of detail on diet.^13^^,^^36^ To determine the quantities of food consumed, we used the Diet Assess food atlas,^33^ which features colour images of eating utensils, dishes, individual foods and meals.

### Food composition database

Another common obstacle for dietary surveys is the lack of a local food composition database.^13^ Our data were analysed using the Balkans Food Composition Database,^23^^,^^33^ which has been developed over the past decade using data from the European Food Information Resource Network,^37^ from the local food industry and from chemical analyses (of mineral and fatty acid content) performed at the Institute for Medical Research in Belgrade, Serbia.^25^ As the local database is a work in progress, before performing our analyses, we supplemented the Balkan database with data from the United States’ Department of Agriculture’s database and incorporated some new foods (e.g. chia seeds), some Bosnian composite dishes and some important nutrients that were missing from existing foods (e.g. docosahexaenoic acid and eicosapentaenoic acid).^38^ In addition, as the Balkans Food Composition Database also incorporates the European Food Safety Agency’s FoodEx2 standardized food classification and description system,^39^ it was possible to compare our survey results with those of other surveys conducted in Europe and to share data with the Global Dietary Database.^36^^,^^40^ Our survey covered all four seasons of the year and data were collected on week days and weekends in the ratio of 70:30. We used Diet Assess software to convert dietary data into data on nutrients and food groups.^25^^,^^41^ Composite dishes were disaggregated into separate food groups and each food item was analysed for nutrient intake, taking into account the food preparation method. Mean nutrient and food intakes were calculated using data from the first survey interview only to stay consistent with other nutrition surveys,^42^ whereas data from both interviews were used to model the usual intake distribution.

### Data collection

As paper-based data collection is slow and relatively expensive, we developed our questionnaires using a Qualtrics computer-assisted personal and telephone interviewing platform with an offline data collection option (Qualtrics, Provo, United States of America).^24^ Interviewers collected dietary, demographic, lifestyle, health and anthropometric data using computer tablets and uploaded the data when an internet connection became available.

Anthropometric measures were self-reported: we provided measuring tapes, scales and instructions to enable interviewees to take measurements in privacy. We validated this approach by verifying waist and hip circumference measurements in around 10% of participants (they were not informed about validation in advance to avoid bias). The intra-class correlation coefficient between the interviewers’ and participants’ measurements was 0.97 (*P* < 0.0001) for waist circumference and 0.91 (*P* < 0.0001) for hip circumference. Blood pressure was measured by the interviewer using an Omron M6 Comfort automated monitor (Omron, Kyoto, Japan) validated for field use in epidemiological studies.^43^^,^^44^

## Initial survey findings

For our survey, four interviewers carried out fieldwork between November 2016 and November 2017 that covered 980 households in 200 enumeration units and collected data on 872 adults (380 men and 492 women). Residents in 24.0% (235/980) of households could not be contacted because they had died or moved or for some other reason, whereas 10.0% (98/980) refused to participate and 66.0% (647/980) participated. Complete data were collected on 96.0% (837/872) of participants and data were lost during transfer from the offline survey application on 3.0% (26/872). In addition, 0.2% (2/872) refused blood pressure measurement, 0.9% (8/872) failed to provide anthropometric measurements and 0.5% (4/872) did not respond to the phone call for the second interview. A systematic error was found in data collected on 19 participants (2.2%) by one interviewer, nutrient values were significantly higher than for other interviewers. Consequently, these observations were excluded from the analysis and the final data sample comprised 853 participants (371 men and 482 women). The final respondents’ profile was representative of the general adult population, excluding pregnant and breastfeeding women (Table 2). The nonresponse rate for households was 34.0% (333/980), which is within the range observed in other dietary surveys.^31^ The initial results show a high estimated prevalence of overweight or obesity (69.0%), abdominal adiposity (63.0%) and hypertension (53.0%).^45^ There were also indications that inadequate intake of some key nutrients and excessive intake of nutrients associated with noncommunicable diseases were common, which underlines the importance of dietary surveillance in this population.

**Table 2:** Dietary survey participants’ characteristics compared with the general adult population, Bosnia and Herzegovina, 2013 and 2017

Characteristic	Adult population^a^ no. (%)	Dietary survey participants,^b^ no. (%)	Difference,%
**Total**	**2 841 794 (100)**	**853 (100)**	**NA**
**Sex**			
Female	1 463 441 (51)	482 (57)	+6
**Age, in years**			
18–40	1 127 597 (40)	241 (28)	−12
41–60	1 033 600 (36)	384 (45)	+9
≥ 60	680 597 (24)	228 (27)	+3
**Geographical location^c^**			
North and central	2 245 017 (79)	586 (69)	−10
South	596 777 (21)	266 (31)	+10
**Place of residence**			
Urban^d^	1 221 971 (43)	373 (44)	+1
**Educational level**			
Total	2 987 440^e^ (100)	853 (100)	NA
Below high school	1 060 840 (36)^f^	266 (31)	−5
High school diploma	1 525 161 (51)^f^	483 (57)	+6
Some further education, including a degree	401 439 (13)^f^	104 (12)	−1

NA: not applicable.

^a^ The 2013 census in Bosnia and Herzegovina recorded a total population of 3 531 159. The adult population aged 18 years and over had to be estimated because the census used the age categories 15 to 19 years and 20 to 24 years.

^b^ The dietary survey conducted in 2017 involved adults aged 18 years or older, excluding pregnant and breastfeeding women and elderly people with cognitive decline.

^c^ These figures were based on the number of inhabitants residing in municipalities categorized as Bosnian (i.e. north and central) or Herzegovinian (i.e. south), a distinction not used in official statistics.

^d^ Urban settlements were those awarded a city status.

^e^ The total population aged 15 years and older.

^f^ Percentage of the population aged 15 years and older.

## Future challenges

During our study, we identified several outstanding issues (Table 1). First, a strategy for finding and retaining reliable and conscientious interviewers is needed to ensure good-quality data are collected. The Institute for Statistics could hire full-time interviewers for multiple surveys rather than recruiting different individuals for each. Second, our use of an outdated sampling frame resulted in a relatively high nonresponse rate and the overburdening of respondents because the same households were repeatedly sampled. The Institute could build long-term relationships with households in the master sampling frame by providing incentives (e.g. gift vouchers) in partnership with local supermarkets. Participants’ details could then be kept up to date and they would be motivated to participate in multiple surveys. Third, because of our household sampling strategy and our use of an outdated sampling frame, we reached our quota of middle-aged women half way through the study but lacked sufficient data on young men. Given financial and time constraints, we changed our strategy and focused on missing age and sex groups. Ideally, future sampling should be based on individuals to avoid this problem. Fourth, our high interviewer costs could be reduced by integrating the dietary survey into the Household Budget Survey as a separate module in a way that does not overburden participants. We were unable to evaluate this approach as our dietary study took place 2 years after the last Household Budget Survey. Fifth, our approach to waist and hip measurement, which showed good correlations between interviewers’ and respondents’ measurements, should be further tested in different population groups as it may be pragmatic in contexts where body contact is inappropriate or where a male interviewer must collect data from female participants. Finally, we did not explore ways of generating a demand for data among users or of encouraging data users to participate in data production, both of which may make dietary surveillance more sustainable.^22^ This was beyond the scope of our study.

In conclusion, we were able to conduct a survey of population dietary habits in a resource-constrained setting with a relatively small investment by relying on an efficient, highly motivated team and by using surveillance structures that commonly exist in many low- and middle-income countries. In particular, collaboration with the national statistics office enabled us to integrate data from multiple surveys and thus identify potential health disparities. The incorporation of dietary surveys into the regular activities of a country’s statistical institutions makes it possible to monitor trends, ideally on a continuous basis. In the future, we plan to extend data collection to children, adolescents and pregnant and lactating women and to foster the participation of data users. We hope our approach can be applied in other low- and middle-income countries to obtain high-quality dietary data for both national and international use.
